# Mycobacterium tuberculosis as a Cause of Periprosthetic Joint Infection After Total Knee Arthroplasty: A Review of the Literature

**DOI:** 10.7759/cureus.4325

**Published:** 2019-03-26

**Authors:** Andrew S Bi, Daniel Li, Yunlong Ma, Decheng Wu, Yuangzheng Ma

**Affiliations:** 1 Orthopaedics, Northwestern University Feinberg School of Medicine, Chicago, USA; 2 Orthopaedics, Second Affiliated Hospital of Guangzhou Medical University, Guangzhou, CHN

**Keywords:** mycoplasma tuberculosis, tuberculosis (tb), revision total knee arthroplasty, total knee arthroplasty, periprosthetic infection, postoperative infection

## Abstract

Total knee arthroplasty (TKA) has become one of the most popular and successful surgeries performed in the world. Infection remains one of the most dreaded complications following TKA, and while rare, tuberculosis as a microbial etiology remains difficult to both diagnose and treat. A review was performed using PubMed, the Cochrane Database of Systematic Reviews, and EMBASE to identify literature pertinent to Mycobacterium tuberculosis infection, TKAs, periprosthetic joint infections, and any combination of the three. The diagnosis of tuberculosis infection after TKA is difficult due to nonspecific signs and symptoms and diagnostic testing. The surgeon should use a comprehensive approach to incorporate the patient’s medical history, physical exam, and blood and imaging diagnostics. Among these, bacterial culture and histopathological examination remain the gold standard of diagnosis, but Polymerase chain reaction technology offers another, more sensitive and rapid option. Treatment strategy centers around on the cornerstone of anti-tuberculosis medical therapy and surgery depending on the clinical situation. While there is a lack of primary literature and standardized guidelines for the diagnosis and treatment of tuberculosis infection after TKA, the overarching principles of the treatment of tuberculosis and the treatment of the periprosthetic infection can be implemented together. There remains room for original research and improvements in both diagnostic testing and treatment.

## Introduction and background

With increasing life expectancy in both developed and underdeveloped countries, the incidence and prevalence of knee osteoarthritis have been steadily increasing [1-3]. In the United States alone, the number of primary total knee arthroplasties (TKAs) is projected to increase by 673%, approximately 3.48 million operations per year [4]. In addition, the average age of surgery continues to decrease, with the demand for primary TKAs in patients less than 65 years old encompassing over 50% of all TKAs [5]. This rise can be attributed to the vast success of the primary TKA, widely considered as one of the most effective procedures of modern surgery [6]. However, a multitude of complications still exist, including but not limited to: resistant pain, hardware loosening, periprosthetic fracture, venous thromboembolisms (VTEs) and infection [7-8].

Infection, in particular, is a dreaded and severe complication after knee replacement surgery. Infection is the most common cause of revision TKA, as the indication for 25.2% of all revision TKAs [9]. The vast majority of infections are caused by typical bacterial organisms, such as Staphylococcus aureus, or Staphylococcus epidermidis; however, Mycobacterium tuberculosis (TB) is a known inciting bacterium that has not been extensively studied. Tuberculosis periprosthetic joint infection (TBPJI) poses a significant burden on the healthcare system, with an average length of hospital stay of 5.1-7.6 days and an average cost of USD 49,360 [9]. Infection not only impacts the patient and the healthcare system, but it also has a drastic effect on surgeons’ own emotions [10]. While the majority of TBPJIs occur within the first two years after surgery, approximately 25% of all TBPJIs occur after two years from primary procedure [11].

First reported by Wolfgang et al. [12] in 1978, TBPJI continues to lack guidelines for diagnosis and treatment. There are many difficulties with early diagnosis of TBPJI, and there are currently no established standards for treatment. The goal of this article is to review and summarize the current body of literature in order to provide a framework for the clinical diagnosis and treatment of TBPJI after TKA.

## Review

Etiology

The cause of postoperative tuberculosis infection can be attributed to one of the two factors: patient factors and operative factors [13]. Patient factors revolve primarily around comorbidities that contribute to an immunosuppressed state: advanced age, obesity, diabetes, autoimmune disorders, large burns, chronic steroid therapy, immunomodulating medications, or retroviral infections [14-16]. The surgery itself can cause an inflammatory response that can both reactivate an old or latent tuberculosis infection, as well as reduce the ability of the immune system to fight off infection.

Postoperative knee joint tuberculosis can be divided into three sources: (1) recurrence of previous tuberculosis infection in a native or postoperative knee; (2) dissemination of tuberculosis from the original source in the lungs or extrapulmonary source; and (3) reactivation of a latent tuberculosis infection [17].

Diagnosis

The diagnosis of tuberculosis infection after TKA is difficult. Harwin et al. [17] reviewed the literature and divided tuberculosis infection after TKA into early and late onset. Early-onset infections were classified as those occurring less than eight weeks after surgery, while late-onset infections were classified as those occurring more than eight weeks post-op. The difficulty of diagnosis originates from the lack of specific clinical symptoms and timely laboratory tests, which often results in delayed diagnosis [18]. Tokumoto et al. [19] believe that there are three reasons for the delayed diagnosis of tuberculosis infection after TKA: (1) a missed diagnosis of joint tuberculosis infection before TKA, (2) suspected tuberculosis infections are difficult to diagnose, and (3) other, more readily diagnosable bacterial infections that can misdirect clinical management. In addition, due to the low pre-test probability of TBPJI, the atypical nature of the presentation, and frequent lack of minor criteria, classic definitions of prosthetic joint infections, such as that of the Musculoskeletal Infection Society (MSIS), can result in false negatives [20]. Currently, the gold standard for diagnosis includes a joint fluid or synovial tissue analysis for acid-fast bacilli culture and histopathological examination [18]. Although the diagnostic specificity is not high, surgeons must consider the patient’s medical history, clinical presentation, as well as imaging findings when approaching their medical decision making.

Medical History

Patients with underlying causes of immunocompromise, history of tuberculosis infection, or patients with tuberculosis risk factors should consider the possibility of postoperative tuberculosis infection prophylaxis [13]. Marschall et al. [21] reported about a 48-year-old male patient with a history of HIV and CD4+ count of 7/uL, who presented with painless swelling of the joint six months after TKA. He was diagnosed with TBPJI at nine months and passed away one month after diagnosis. The tuberculosis was found to be disseminated throughout multiple organ systems.

Local or Systemic Symptoms and Signs

When a TKA is complicated by TBPJI, local symptoms of the knee are mostly inflammatory reactions such as pain, swelling, and fever. More severe signs can manifest in a small proportion of the population, such as purulent drainage, abscess or sinus formation. Harwin et al. [17] reported on a 60-year-old female patient who developed a 4 cm by 4 cm painful mass on the medial side of the proximal metatarsal, seven months after TKA. The mass gradually enlarged, and surgical exploration demonstrated a cheese-like granulation tissue on the inside of the mass.

In patients with local tuberculosis infection after TKA, the classic systemic symptoms of tuberculosis infection such as weight loss, fatigue, or night sweats are rare. In these patients, the only other presenting symptom besides local symptoms of TBPJI can be a second local infection, i.e. of the lungs, or a second joint. Wang et al. [22] reported on a 72-year-old male patient with a 50-year smoking history who developed a chronic cough and progressive knee pain one year following a TKA. Sputum cultures and knee synovial tissue culture confirmed the diagnosis to be TBPJI and pulmonary tuberculosis infection. Bryan et al. [23] reported about the case of a 72-year-old female with knee pain and swelling after TKA, along with a painful, swollen elbow. She denied any history of tuberculosis infection, yet histological examination found caseous granulomas, confirming the diagnosis. Other complications secondary to contiguous spread can present, such as local bone destruction, osteomyelitis, and prosthetic loosening.

Blood Diagnostic Testing

Laboratory tests from plasma for TBPJI have extremely low specificity [24]. Oftentimes laboratory results can be within normal reference ranges. When abnormalities do result, they usually include an elevated white blood cell count, or increased inflammatory markers, such as erythrocyte sedimentation rate and C-reactive protein. However, these hematologic indicators have poor specificity, acting as markers of general inflammatory activity rather than indicating the patient has an infection by Mycobacterium tuberculosis [25]. One blood test of note would be the QuantiFERON-TB Gold^©^, which is an interferon-gamma release assay (IGRA). However, due to the limitation of measuring a surrogate marker of tuberculosis, it cannot distinguish between latent and active infection, and thus its usefulness in the acute setting is limited [26].

Bacterial Culture and Histopathological Examination

Acid-fast bacilli (AFB) staining and cultures, along with a histopathological examination of synovial tissue demonstrating caseating granulomas remains the gold standard for the diagnosis of Mycobacterium tuberculosis [27]. However, even with these methods, there can be false negatives in testing. If the surgeon’s tissue biopsy misses an area of active infection, or if a bacterial load is too low to culture on Löwenstein-Jensen medium, false negatives can result. In addition, the growth of tuberculosis in cultures often takes weeks, with an average of 23.7 days [28]. Besser et al. [29] reported on a primary TKA case for osteoarthritis, in which synovial hyperplasia and erythema were observed during operation. Biopsy of the tissue returned positive for tuberculosis infection. Marmor et al. [30] reported that a 66-year-old male developed knee pain and swelling two months after right TKA. Doppler ultrasonography showed an abscess in the right popliteal fossa and was subsequently surgically removed and cultured. While the results of the joint and abscess cultures were negative, the AFB were finally isolated from blood samples on acid-fast staining and culture. An example of a case that emphasizes the importance of multiple tissue samples comes from Wray et al. [31], in which diagnosis of tuberculosis infection was made not with direct tissue biopsy, but rather sputum culture in conjunction with clinical symptoms, allowing a 63-year-old male to receive early directed therapy for TBPJI.

Polymerase Chain Reaction Technology

Polymerase chain reaction (PCR) technology has recently changed the paradigm for the diagnosis of tuberculosis. It has the advantages of rapid turnaround, increased sensitivity, and ability to detect trace loads of bacteria, causing a higher percentage of diagnoses to be made with PCR in recent years [24]. Some studies have shown PCR can even detect tuberculosis infection from tissue samples of purulent material or granulation tissue in TBPJI [32]. In testing respiratory samples, sensitivity and specificity are 87.1% and 99.9%, respectively, and when testing extrapulmonary specimens, sensitivities range from 53.7% to 100% depending on sample selection. Neogi et al. [33] reported on a 73-year-old female who developed TBPJI 14 years after a TKA with negative synovial tissue and joint fluid cultures. Ultimately, PCR of synovial tissue detected Mycobacterium tuberculosis.

Imaging 

Imaging for the diagnosis of tuberculosis infection is often focused around the lungs, with chest radiographs and non-contrast computed tomography (CT) scans the primary focus for active vs. latent infection [34]. Imaging for extrapulmonary tuberculosis has been less extensively studied. Imaging findings in musculoskeletal tuberculosis infection, in particular, are often nonspecific and only indicate a non-specific infectious etiology [35]. In the initial stages of the disease, plain radiographs can show widening of the joint space with soft tissue swelling. With further disease progression, marginal erosions, blurring of the articular cortex, and loss of subchondral bone can be seen [35]. A classic constellation of findings was described in Phemister’s triad: juxta-articular osteoporosis, peripherally located osseous erosions, and gradual narrowing of the joint space [36]. The use of CT and magnetic resonance imaging (MRI) is limited secondary to artifact produced from the metal implant, a problem not unique to TBPJI. Nuclear medicine techniques have been found to be sensitive but not specific [34-35]. Zeiger et al. [37] and Spinner et al. [38] reported on the use of radionuclide scanning to show that the knee joint of surgical sites had increased absorption of tracer (99mTc-MDP, etc.), but was not specific to the diagnosis of tuberculosis infection.

Treatment strategy

There are currently no established standards in managing patients with TBPJI. Key principles are source control, anti-tuberculosis medications, and early diagnosis and treatment, or empiric therapy in cases when a diagnosis might be delayed. Wray et al. [31], reported on a case in which, after developing fever and knee pain following a primary TKA, a 63-year-old male patient was empirically put on anti-tuberculosis treatment. Histopathologic diagnosis followed a few weeks later, and a year after surgery the knee joint had recovered without the need for revision surgery. However, in most cases, the general treatment paradigm involves anti-tuberculosis drug therapy in addition to a thorough joint debridement. Depending on the timing and severity of infection, the prosthesis can occasionally be preserved, with revision surgery and fusion providing alternative options [17].

Anti-tuberculosis Medical Therapy

According to the Infectious Disease Society of America (IDSA), recommended medical treatment regimens for extrapulmonary tuberculosis are similar to the pulmonary disease. This consists of initiation of two months of “RIPE” therapy; rifampin, isoniazid, pyrazinamide, and ethambutol, followed by six to nine months of rifampin and isoniazid [39]. On occasions when TBPJI occurs early, within six to eight weeks, standard anti-tuberculosis medical therapy alone is possible. However, this treatment regimen requires not only rapid diagnosis, but also no other sites of infection, prosthetic loosening, or osteolysis. There are scant reports of late-onset TBPJIs being successfully treated with medical therapy, but the majority of current literature supports surgical intervention in addition to anti-tuberculosis treatment [40].

Anti-tuberculosis Medical Therapy, Joint Incision and Drainage (I&D), and Preservation of Prosthesis

The option of treating TBPJI with I&D and medical therapy while retaining the prosthesis is mainly limited to the following clinical situations: (1) early-onset infection; (2) clinical and imaging findings consistent with an absence of prosthetic loosening; and (3) sensitivity to anti-tuberculosis medications [41]. Multiple case reports have demonstrated success in treating the infected joint with extensive lavage and antibiotics [29,31]. These all cases included a diagnosis within eight weeks following primary surgery, and an extended course of medical therapy greater than nine months, allowing the original prosthesis to be preserved.

Anti-tuberculosis Medical Therapy, Joint I&D, and Two-Stage Replacement Arthroplasty

Nearly all late-onset TBPJI, severe suppurative infections, active sinus tract formation, signs of periprosthetic osteolysis or prosthetic loosening, should undergo a two-stage replacement arthroplasty, as results have been shown to be as effective as 90% [42]. Patients must be medically fit for multiple surgeries and have adequate bone stock, but this two-stage revision remains the gold standard for a late-onset joint infection following arthroplasty [43]. Spinner et al. [38] reported on a 70-year-old female who had recurrent suppurative sinus tract formation following a primary TKA. She was later diagnosed with tuberculosis infection, and after multiple debridements, underwent a two-stage revision along with anti-tuberculosis medications for 12 months. The patient did well, without a need for further revision or fusion. Khater et al. [44] reported on a 75-year-old female that was found to have a 2-3 mm wound in the surgical incision site three months after a right primary TKA. Histopathologic testing revealed tuberculosis infection, and a two-stage revision was performed using vancomycin and rifampin spacers. After 18 months of anti-tuberculosis medications, there was no recurrence of TBPJI. These are just a few examples of successful cases of treatment of late-onset TBPJI.

Anti-tuberculosis Treatment, Removal of Prosthesis and Arthrodesis

Knee arthrodesis is a procedure resulting in severe limitation of functionality and quality of life. Nearly all arthrodeses are performed secondary to failed knee arthroplasties, with the most common indications due to PJI, extensor mechanism disruption, soft-tissue deficiency, and severe bone loss [45-47]. Carrega et al. [48] reported on multiple patients who developed recurrent TBPJI after primary TKA and subsequent revision surgeries. Biopsy of infected tissue confirmed tuberculosis infection, and following debridement, the patients underwent arthrodesis and extensive courses of anti-tuberculosis drug therapy. There were no recurrences, and the patients were able to ambulate a year after surgery. While arthrodesis is not a first-line treatment option, it can be a useful option for patients with persistent infection and medical comorbidities limiting the ability to tolerate multiple procedures [49].

## Conclusions

TB is a rare etiology of PJI after primary TKA, and diagnosis requires a high level of pretest suspicion along with a knowledge of the various non-specific signs and symptoms. Bacterial AFB culture and histopathological examination continue to be the current gold standard for diagnosis; however, modern PCR technology has improved to become a useful tool in the surgeon’s arsenal for the diagnosis of TBPJI. There is a vast room for future research to improve the standardization and systemization of the diagnosis paradigm in attempts to improve not only the sensitivity and specificity but also the speed and rapidity of diagnosis. The current treatment regimen continues to center around the classic anti-tuberculosis initial quadruple therapy. Medical therapy alone has rare and limited indications and is relegated primarily to mild cases of early-onset post-operative infections. The key treatment divergence centers around retention or replacement of the original prosthesis. Often times prosthetic aseptic loosening or persistent infection are the core factors which influence the decision-making process. For late-onset infections, the current gold standard of treatment is similar to those of other PJIs with more common microbes. Two-stage revision arthroplasty can eradicate the infection and provide higher quality functional outcomes, but in difficult cases, arthrodesis or even amputation is occasionally necessary. The exact choice of treatment for each patient should be specifically tailored to the clinical scenario, but future work needs to be done in analyzing quantitative outcome data following the various types of treatment. Due to the lack of current literature, case reports and case series continue to be a useful source of primary data and should be encouraged to be published with standardized reporting with a focus on thorough patient demographics and evidence-based outcome data.
